# Transcription factors as essential regulators of myometrial smooth muscle cell function during pregnancy and labor

**DOI:** 10.1007/s00018-026-06341-8

**Published:** 2026-07-21

**Authors:** Sabrina Martini, Palak Gujral, Eduardo Orozco-Alonso, James Saliba, Volker Blank

**Affiliations:** 1 Lady Davis Institute for Medical Research, 3755 Côte Sainte-Catherine Rd, Montreal, QC H3T 1E2 Canada; 2 Department of Medicine, McGill University, Montreal, QC H4A 3J1 Canada; 3 Department of Physiology, McGill University, Montreal, QC H3G 1Y6 Canada

**Keywords:** Transcription factor, Myometrium, Cytokines, Pregnancy, Labor, Preterm

## Abstract

Parturition is a tightly regulated process coordinated by myometrial smooth muscle cells, which generate the uterine contraction required for birth. Preterm labor, defined as labor occurring before 37 weeks of gestation, remains a significant clinical challenge due to its association with adverse short and long-term risks on neonatal and maternal health. Earlier and recent studies provide strong evidence that transcription factors are performing crucial functions in uterine smooth muscle cells. In this review, we focus on the PR/ER, PPAR, NF-κB, AP-1, NFE2L2, and MAFF transcription factors and provide a comprehensive overview of their respective roles in myometrial smooth muscle cell function and the labor process. We also briefly discuss other transcriptional regulators that have been linked to uterine quiescence during pregnancy, to activation of the labor process or to both functions. Collectively, these transcription factors control various cellular processes, including inflammation, regulation of cytokine, chemokine and metalloproteinase expression, oxidative stress response, prostaglandin synthesis, calcium signaling, and contractility. Monitoring and targeting these transcription factors and associated pathways in the uterus are key for delaying or preventing preterm labor through the design of novel diagnostic and therapeutic tools to improve birth outcomes.

## Introduction

Pregnancy is a complex physiological process that leads to labor and birth through tightly regulated events that ensure coordinated activation of uterine contractility, cervical remodeling, and fetal membrane rupture [1]. Throughout gestation, the uterus remains in a quiescent state, despite progressive mechanical stretch and endocrine changes. The transition to labor is marked by activation of the myometrium, the smooth muscle layer of the uterus, resulting in synchronized contractions that drive cervical dilation and delivery of the fetus [2]. Hence, successful parturition depends on precise control of molecular and cellular pathways regulating uterine structure and function. These processes are regulated by complex endocrine, inflammatory, metabolic, and mechanical signals acting across maternal and fetal compartments. Hormones, cytokines, lipids, and enzymes cooperate to establish uterine quiescence during pregnancy and to promote activation at term, with transcription factors serving as key regulators of these signals by orchestrating their expression or synthesis [3–5]. Normal labor and birth occur between 37 and 42 weeks of pregnancy, but a series of conditions and risk factors, including infection, chronic conditions, such as diabetes, or stress may lead to early onset of contractions [6].

Preterm birth is defined as delivery occurring prior to 37 weeks of pregnancy. It has been estimated that 13.4 million infants, about 9.9% of all births, were born preterm worldwide in 2020 [7, 8]. Despite advances in obstetric and neonatal care, preterm birth continues to carry major risks for both infants and mothers. Surviving premature newborns often face long-term complications across multiple systems, including neurodevelopmental impairment, increased cardiovascular risk, chronic lung disease, renal dysfunction, metabolic disorders; and potentially elevated risk for childhood cancers [9–11]. A link between infection/inflammation and preterm labor has been firmly established [12]. Infection of the uterus or amniotic fluid by bacteria, viruses, fungi, and parasites accounts for a minimum of 25–40% of preterm births [13, 14]. However, a large proportion of preterm labor arises from sterile inflammation in the absence of pathogens [15, 16]. In both settings, premature activation of immune pathways promotes the early production of proinflammatory mediators.

Although the precise mechanisms involved in labor initiation remain incompletely defined, a substantial amount of evidence supports a central role for inflammatory processes. Labor is driven physiologically by an interplay between hormonal and mechanical factors, and pathologically, it has been linked to infection, bleeding, and excessive uterine stretch [17, 18]. At term, the inflammatory response of labor involves an influx of neutrophils and macrophages into the uterine smooth muscle layer and cervix [19–21]. Cytokines play critical roles in parturition, cervical dilation, rupture of membranes, and uterine contractility. Cytokine release induces activation of phospholipid metabolizing enzymes and extracellular matrix (ECM) remodeling enzymes, leading to prostaglandin (PG) secretion and matrix metalloproteinase (MMP) activation, resulting in preterm labor [22–26]. The inflammatory response is defined by an increase of various proinflammatory cytokines and chemokines, such as interleukin-1 beta (IL1B), interleukin 6 (IL6), C-X-C motif chemokine ligand 8 (CXCL8), and tumor necrosis factor (TNF, formerly TNF-α), within different biological compartments including the amniotic fluid, myometrium, decidua, fetal membranes, and maternal serum [22, 25–28]. Elevated proinflammatory cytokines in the amniotic fluid are strongly associated with vaginal and cervical infections. This suggests a function for cytokines in the onset of preterm labor, regardless of the presence of infection. The high levels of proinflammatory cytokines found in uterine tissues in infection-induced preterm labor is likely to originate from infiltrating leukocytes or decidual, myometrial, amnion, and placental cells [20]. It has also been shown that the physiological balance between reactive oxygen species (ROS) and antioxidants is essential throughout pregnancy. Uterine smooth muscle contraction, the hallmark of labor, is associated with oxidative stress. Elevated ROS are implicated in preterm birth, preeclampsia, and gestational complications [29–33]. Preterm or term labor induction involves a complex interplay between inflammation and oxidative stress, which must be tightly regulated to ensure a successful pregnancy outcome [34].

Over the past decades, multiple transcription factors have been identified as key regulators of pregnancy maintenance, myometrial quiescence, and activation of labor and birth [5, 35–42]. However, the regulatory networks and associated signaling pathways governing these processes have not been fully elucidated. In this review, we focus on the PR/ER, PPAR, NF-κB, AP-1, NFE2L2, and MAFF transcriptional regulators, which were selected based on the following criteria: (i) strong evidence linking them to gestation and labor, (ii) representation of major biological axes involved in parturition – including endocrine signaling, inflammation, metabolism, oxidative stress, and contraction – and (iii) their potential translational relevance as potential biomarkers or therapeutic targets. Together, they provide representative examples of how distinct signaling pathways converge at the transcriptional level to regulate pregnancy progression and labor onset. Nevertheless, additional transcription factors have been shown to function as drivers of pregnancy and parturition, we will thus introduce these briefly to provide a more comprehensive overview and to encourage research in these areas.

The myometrium consists of multiple cell types, with the main component being myometrial smooth muscle cells, but the tissue also comprises immune cells, endothelial cells, fibroblasts and nerve fibers. In this review, we are focusing on the regulation of myometrial smooth muscle cells. We refer to these uterine myocytes also simply as myometrial cells.

## Transcription factors, myometrium and labor

Transcription factors regulate gene expression by binding specific DNA regions and modulating the transcriptional activity of target genes. This review will focus on transcription factors hypothesized to act as vital players in the regulation of the myometrium and the labor process during pregnancy.

### PR and ER

The hormone progesterone is widely known for its involvement in various reproductive processes including the menstrual cycle, ovulation, pregnancy establishment and maintenance, parturition, and lactation [43–45]. In most mammals, labor is triggered by a decrease in circulating progesterone levels which abrogates its inhibitory effects on uterine smooth muscle cells [43]. However, in humans, labor occurs in the presence of elevated circulating levels of progesterone which gave rise to the alternate theory of “functional progesterone withdrawal”. This hypothesis proposes that human myometrial cells develop a decreased responsiveness to PR-mediated progesterone actions [46]. Thus, the onset of labor is linked to the inhibition of progesterone signaling whether it be via decreased progesterone levels in most mammals or decreased responsiveness to progesterone in humans.

**Fig. 1:**
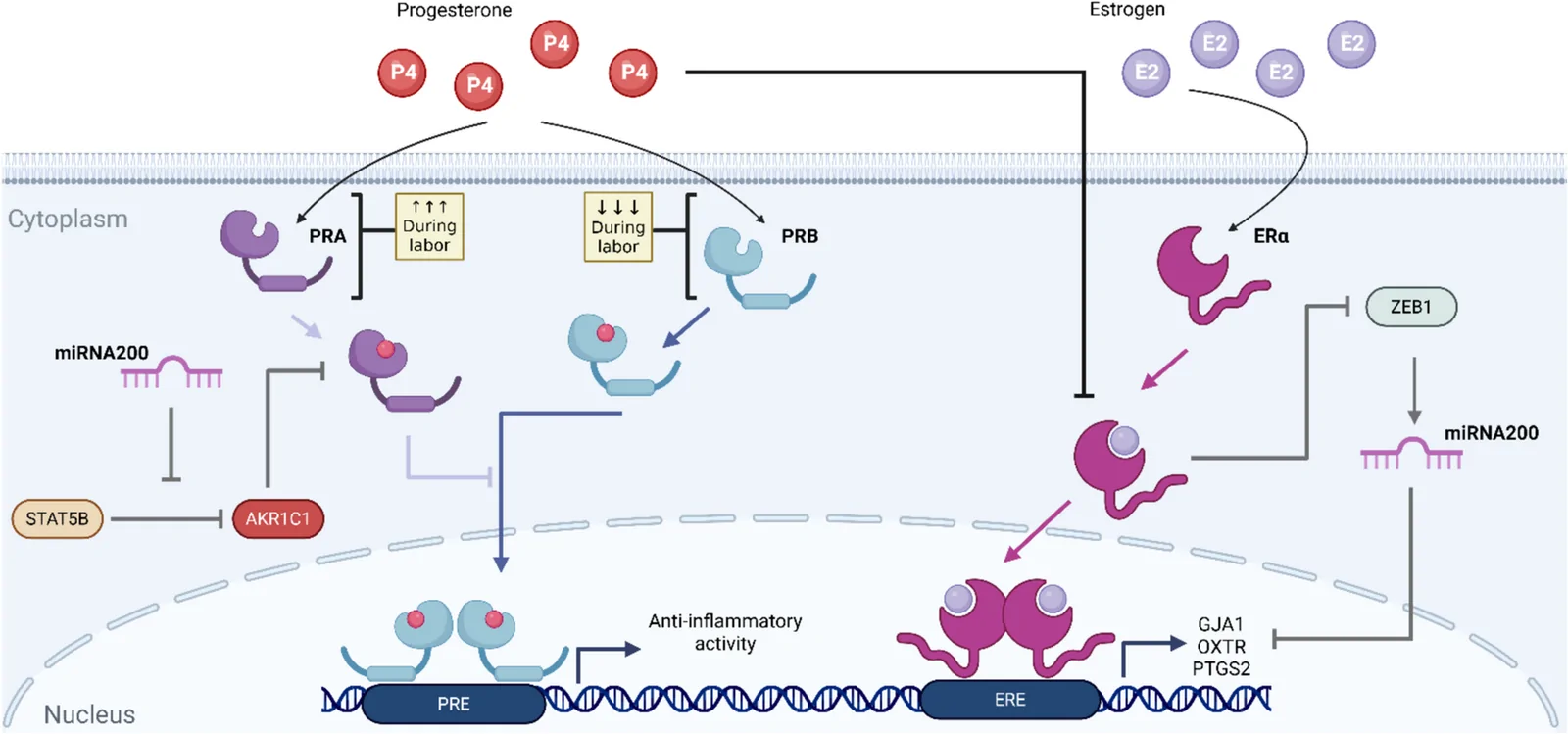
**Progesterone receptor and estrogen receptor signaling pathway in the transition from uterine quiescence to activation. **Increased PRA and decreased PRB levels shift progesterone signaling, reducing PRB mediated anti‑inflammatory transcription and contributing to functional progesterone withdrawal in myometrial cells. ERα signaling promotes a contractile myometrium by upregulating the expression of contraction-associated proteins (CAPs) such as GJA1, OXTR, and PTGS2. The miRNA200 family inhibits the expression of genes such as STAT5B and CAPs to promote quiescence and inhibit contractility. Created with BioRender.com

Progesterone exerts its effects primarily through two isoforms of the progesterone receptor, PR (Fig. 1), namely PRA and PRB, which are ligand-activated transcription factors that belong to the steroid superfamily [47]. PRA is a truncated form of PRB, lacking the first 164 N-terminal amino acids [47, 48]. The “PRA: PRB hypothesis” proposes that functional progesterone withdrawal for initiation of human labor occurs through an increased PRA: PRB ratio, with low PRA levels during pregnancy and significantly increased PRA expression in the laboring uterus [49–51]. A recent study provided mechanistic insights, showing that changes in AP-1 protein composition contribute to functional progesterone withdrawal, as AP-1 dimers form a complex with PR. Due to the switch from JUN/JUN to FOS/JUN during labor [52], their affinity to PR isoforms changes from PRB during pregnancy to PRA during labor, altering PR function from being a transrepressor to a transactivator of the contractility-associated gap junction alpha 1 (GJA1) gene, also known as connexin-43 (Cx43). The study also found that PRB is liganded during gestation, but PRA becomes unliganded during labor due to increased levels of the progesterone-metabolizing enzyme aldo-keto reductase family 1 member C1, AKR1C1 (20α hydroxysteroid dehydrogenase), resulting in reduced nuclear progesterone levels [52]. Another report suggested that the increase in AKR1C1 is mediated by the microRNA-200 family via repression of STAT5B (signal transducer and activator of transcription 5B), a repressor of the enzyme [53]. MicroRNAs (miRNAs) are small non-coding RNA that have been shown to inhibit uterine contractility by downregulating the transcription of contraction-associated proteins (CAPs) [54, 55]. In the myometrium, miRNAs typically inhibit gene expression to regulate myometrial quiescence and contractility. Furthermore, it has been shown that proinflammatory signals such as lipopolysaccharide (LPS) in preterm labor induce AKR1C1 transcript and protein levels in myometrial cells [56]. This upregulation is mediated by AP-1 and NF-kB transcription factors and results in the induction of contraction-associated protein GJA1. It was also found that, in human myometrial cells, PR increases the expression and decreases the degradation of IκB, a kinase known to inhibit proinflammatory NF-kB signaling [57]. This suggests a link between uterine inflammation and local progesterone withdrawal at the onset of labor. A more recent analysis revealed that tissue resident M1 macrophages constitute a source of interleukin-1 alpha (IL1A), which promotes intracellular progesterone metabolism and contributes to uterine activation at labor onset [58]. Together, these studies confirm that the balance of progesterone receptors is critical for the initiation of labor.

Progesterone receptor signaling in the myometrium cannot be fully understood in isolation. It is important to consider estrogen receptor (ER), another major transcriptional regulator of myometrial function, as crosstalk exists between PR and ER pathways. Although four forms of estrogen exist, the word ‘estrogen’ is commonly used as a synonym for estradiol (E2) or more specifically, 17β-estradiol [59]. This is because estradiol is the most predominant form of estrogen intracellularly. Hereafter, “estrogen” will be used to refer to estradiol. Estrogen transcriptional action is mediated through ERα and ERβ, encoded by the *ESR1* and *ESR2* genes, respectively. This review will focus on ERα as several studies have demonstrated that it is the dominant ER isoform controlling the myometrium in pregnancy and labor [60, 61]. ER isoforms bind estrogen, homodimerize, translocate to the nucleus, and function as transcription factors by binding DNA at estrogen responsive elements (EREs) on promoters of estrogen responsive genes. Estrogen promotes a pro-contractile phenotype in the uterus by inducing the expression of contractility associated proteins such as GJA1, oxytocin receptor (OXTR), and prostaglandin-endoperoxide synthase 2 (PTGS2) [60, 62–64]. Recent evidence has shown that ERα activation suppresses zinc finger E-box binding homeobox 1 (ZEB1)-mediated expression of the anti-inflammatory miRNA200, which allows PTGS2 upregulation in human myometrial cells. Lower levels of ZEB1 also allowed for de-repression of GJA1 and OXTR, both of which induce uterine contractility, leading to labor [53]. Thus, for the myometrium to transition from a quiescent to a contractile state at the time of parturition, functional progesterone withdrawal is accompanied by “estrogen activation”, where circulating estrogen levels are increased to enhance ER signaling and downstream CAP expression. In fact, progesterone has been shown to attenuate ERα expression in human myometrium [65], hamster uterine tissue [66, 67], and rhesus monkey uterine tissue [68], thus supressing ER signaling and maintaining uterine quiescence. Therefore, the transition to labor not only reflects functional progesterone withdrawal, but also a release of progesterone-mediated inhibition of ER signaling, which results in unopposed estrogen action and myometrium activation.

## PPAR

**Fig. 2:**
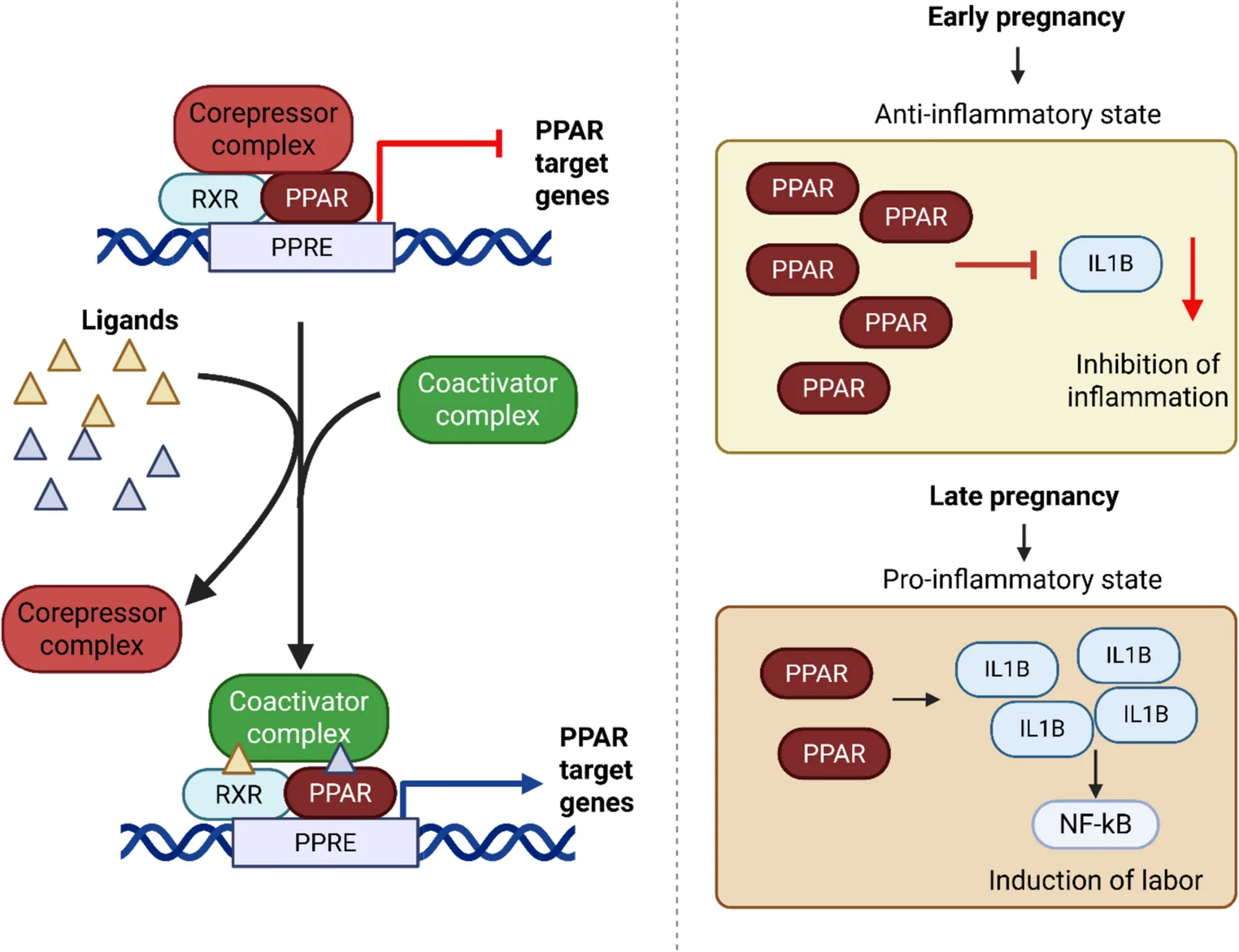
**PPAR signaling pathway in pregnancy and labor. **Left: Ligand binding releases corepressors from PPAR-RXR complexes, allowing binding of coactivators and transcriptional activation through PPREs. Right: In early pregnancy, high PPAR activity supports an anti-inflammatory environment, whereas in late pregnancy, reduced PPAR activity permits elevated IL1B and NF-κB signaling, contributing to the pro-inflammatory state associated with labor onset. Created with BioRender.com

Peroxisome proliferator-activated receptors, PPARs (Fig. 2), are transcription factors and members of the nuclear hormone receptor superfamily. There are three major isoforms of PPAR: PPARA (PPARα), PPARD (PPARβ/δ), and PPARG (PPARγ) [69]. These transcription factors control genes involved in lipid metabolism, particularly fatty acid oxidation. PPARs are activated by binding of specific ligands like long-chain polyunsaturated fatty acids, such as arachidonic acid and linoleic acid, where arachidonic acid is the precursor for prostaglandins [70–72]. PPARs function as heterodimers with retinoid X receptor (RXR), binding DNA at PPAR response elements (PPREs) located in the promoter region of target genes [70, 73–75]. In the absence of a ligand, the heterodimer recruits corepressors such as nuclear receptor corepressor (NCoR) and silencing mediator of retinoic acid and thyroid hormone receptor (SMRT), together with histone deacetylases (HDACs), to maintain transcriptional repression [76–79]. Ligand binding induces a conformational change that displaces corepressors and promotes recruitment of histone acetyltransferase (HAT)-containing coactivators, activating target gene transcription [70, 73, 74, 80–83].

A study revealed that PPARA expression in human myometrium changes during pregnancy and labor, being significantly upregulated in preterm not in labor specimens and markedly downregulated in term labor samples [84]. A previous report had already shown that PPARA expression is reduced in the choriodecidua with the onset of labor in normal pregnancy [85]. The beginning of labor is characterized by a transition from uterine quiescence to active contractility, accompanied by a shift from an anti‑inflammatory to a proinflammatory environment [86]. Further research has shown PPARA-deficient mice exhibit increased IL6 secretion [87]. PPARA activity also inhibits MMP9 expression and secretion, favoring the maintenance of uterine quiescence, as MMPs are associated with uterine contractility [88]. Hence, increased PPARA expression plays a critical role in early and mid-pregnancy by maintaining an anti-inflammatory cytokine environment and suppressing labor. Consequently, at the onset of labor, PPARA expression in the myometrium is found to be significantly decreased, correlating with increased expression of IL1B, which promotes uterine contractions [84].

Downregulation of PPAR levels has also been associated with preterm labor, particularly in infection-driven preterm births, as an untimely inflammatory response is triggered, stimulating labor. Dysregulation in PPARG signaling was shown to be associated with preterm labor [89]. Another investigation revealed that activating the anti-inflammatory PPARG pathway with rosiglitazone, a PPARG agonist, reduced the incidence of preterm birth in a mouse model of inflammation-induced preterm birth [90]. Rosiglitazone aids in reducing inflammation by inhibiting NF-κB activity in both myometrial and decidual macrophages. Rosiglitazone also exhibits antioxidant effects, as further studies showed that rosiglitazone treatment leads to elevated expression of the antioxidant transcription factor NFE2L2 and its downstream target HO-1 in macrophages, while simultaneously downregulating toll-like receptor 4 (TLR4) [91]. TLR4 activation has been shown to induce ROS production, leading to oxidative stress [92]. Oxidative stress is a natural part of pregnancy, and a balance between ROS and antioxidant production is crucial to limit complications. Excess ROS is linked to oxidative stress damage and induces preterm labor [93]. Hence, PPARs are crucial regulators of the inflammatory and oxidative stress pathways, and their activation may help to prevent preterm birth.

## NF-κB

**Fig. 3:**
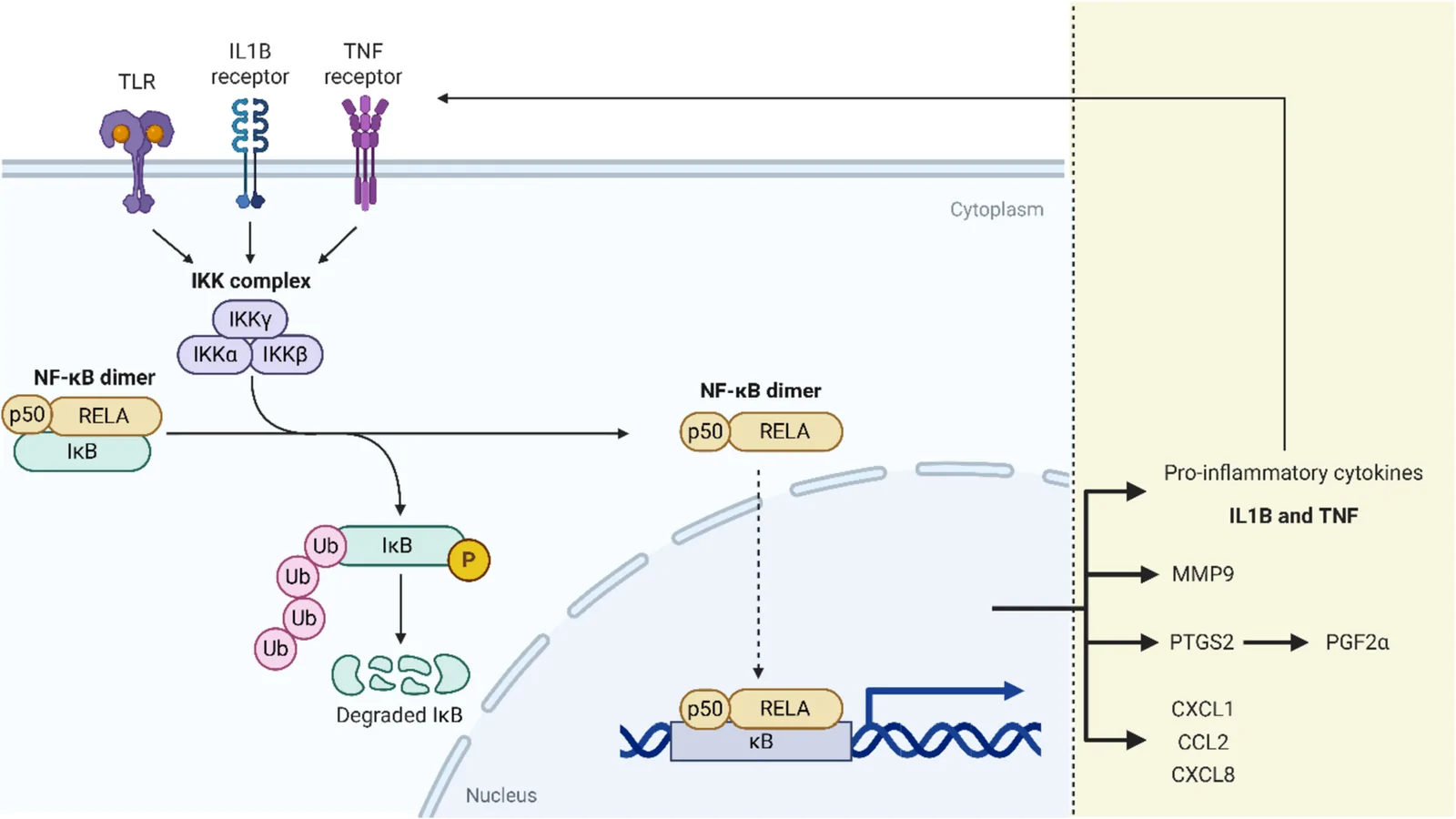
**NF-κB signaling pathway in term and preterm labor. **Activation of the NF‑κB pathway by TLR, IL1B, and TNF receptors. Ligand stimulation activates the IKK complex, leading to IκB phosphorylation, ubiquitination, and degradation. The released NF‑κB (p50/RELA) dimer translocates into the nucleus to induce transcription of pro‑inflammatory cytokines (IL1B, TNF), MMP9, PTGS2–mediated PGF2α production, and chemokines (CXCL1, CCL2, CXCL8), promoting uterine inflammatory activation. Created with BioRender.com

Nuclear factor kappa B, NF-κB (Fig. 3), is a family of transcription factors that control the expression of genes involved in immune responses and inflammatory pathways. It was initially discovered in 1986 when it was recognized as a regulator of kappa light chain expression in murine B cells [94]. Since then, NF-κB has been identified in many cell types. The NF-κB family consists of proteins belonging to the REL family, which includes RELA subunit p65, RELB, c-REL, p105/p50 (NFKB1), and p100/52 (NFKB2) [95, 96]. These proteins are homologous in a sequence within the N-terminal region called the Rel homology domain (RHD), which mediates the dimerization, DNA binding and interaction with inhibitory proteins [95, 97, 98]. NF-κB is normally retained in the cytoplasm in an inactive state by being bound to an inhibitory protein, IκB. NF-κB can be activated by proinflammatory cytokines such as IL1B and TNF or by LPS binding their respective receptors [99]. LPS, for instance, binds and activates Toll-like receptor (TLR)-4 on the cell surface [100]. In response to these stimuli, the IκB kinase (IKK) complex is activated, and IκB is phosphorylated, ubiquitinated, and targeted for degradation, thereby allowing NF-κB to translocate into the nucleus, bind DNA and regulate target gene expression [96, 99]. In addition to being activated by proinflammatory cytokines, NF-κB regulates these proinflammatory mediators in human gestational tissues such as the placenta, amnion and choriodecidua. The κB motif that NF-κB binds to on the DNA has been identified in the promoter regions of several proinflammatory cytokines such as TNF, IL6, and IL8 [101]. In the case of infection-induced preterm labor, LPS activates NF-κB which enhances TNF, IL1B, and IL6 synthesis and release. Because these proinflammatory cytokines can activate NF-κB as well, a positive regulatory loop exists in labor which is thought to amplify uterine inflammatory responses. It is thought that NF-κB activity is suppressed during pregnancy and elevated at the onset of labor [36]. Indeed, it has been shown that localization of the RELA subunit in the nucleus of myometrial cells is significantly associated with term and preterm labor [102]. Thus, NF-κB is involved in promoting labor by activating transcription of proinflammatory genes while also being itself activated by inflammatory stimuli.

NF-κB is also involved in prostaglandin synthesis. Arachidonic acid is the precursor for prostaglandins, such as PGF_2α_ and PGE_2_, which mediate cervical ripening, dilation, and myometrial contractions, leading to the onset of labor [103]. Prostaglandin-endoperoxide synthase, PTGS (formerly cyclooxygenase, COX), is the rate-limiting enzyme in prostaglandin synthesis. Two isoforms of PTGS exist: PTGS1, which is constitutively expressed, and PTGS2, which is induced by proinflammatory cytokines such as IL1 and TNF. An earlier study showed that NF-κB mediates IL1B induced PTGS2 expression in human myometrial cells [104]. Treatment of human myometrial cells with IL1B led to the degradation of IκB which induced nuclear translocation and binding of NF-κB to DNA. This results in upregulation of PTGS2 transcript and protein levels and consequently elevated prostaglandin production. IκB phosphorylation alone is not sufficient for the release and activation of NF-κB [105]. IκB must be degraded via proteolysis to dissociate from NF-κB and induce subsequent NF-κB nuclear translocation and activity [105, 106]. Calpain I inhibitor blocks the degradation of IκB by inhibiting cysteine proteases but does not block IκB phosphorylation [105]. Thus, inhibiting IκB degradation with calpain I inhibitor prevents NF-κB nuclear translocation and decreases PTGS2 expression and prostaglandin synthesis [104]. These findings show that NF-κB signaling directly regulates prostaglandin production, which are known inducers of myometrial contractions during labor [104]. A more recent study revealed that PGF_2α_ itself activates RELA and PTGS2 in human myometrial cells, indicating a positive feed-forward loop at the time of labor [107]. Additional experiments from the same study also concluded that PGF_2α_ induces calcium/cAMP-dependent transcription factors, including cAMP response element-binding protein (CREB) and CEBPB CCAAT/enhancer-binding protein beta (CEBPB), involving calcium-dependent signaling through GNAQ (G protein subunit alpha q) and GNAI (G protein subunit alpha i) proteins [107]. NF-κB thus acts as a potent catalyst for labor as it induces prostaglandin production, which in turn will further activate NF-κB [104, 107]. Early or abnormal activation of NF-κB may thus play a role in inducing preterm labor.

Chemokines, a class of cytokines that act as migration guides, play an important role in attracting and recruiting immune cells. Experiments showed that stretch stimulation, as well as IL1B and TNF treatment in primary myometrial cells correlated with an increase in *CXCL1*, *CCL2*, and *CXCL8* chemokine mRNA expression in term and preterm labor samples [108]. This increase in chemokine expression was reported to be due to NF-κB signaling as RELA overexpression drives chemokine expression [109]. These findings suggest that NF-κB contributes to preterm labor by inducing chemokine expression, which in turn leads to the recruitment of inflammatory immune cells in the myometrium. The recruited immune cells then release proinflammatory cytokines, further activating NF-κB and amplifying chemokine production. As a result, a positive feedback loop develops, escalating inflammation and leading to the onset of labor [108].

Matrix metalloproteinases (MMP) are enzymes involved in the degradation and remodeling of the extracellular matrix (ECM). During the process of labor, these MMPs, mainly MMP2 and MMP9, contribute to cervical ripening, rupture of fetal membranes, and uterine contractions [110–113]. Adding purified MMP2/9 to myometrial tissue strips enhanced oxytocin-induced contractility, while their suppression using the MMP2/9 inhibitor SB-3CT resulted in relaxation [114, 115]. Inhibition of NF-κB binding activity leads to the repression of LPS-stimulated MMP9 activity in human fetal membranes [36, 116]. Furthermore, the *MMP9* gene locus comprises regulatory regions containing NF-κB recognition elements. This suggests that *MMP9* transcription is regulated directly by NF-κB and is involved in fetal membrane rupture [117]. Another study demonstrated that NF-κB activation by IL1B in human term myometrium correlates with increased MMP9 production [118]. Thus, myometrial activation mediated by NF-κB may also contribute to tissue remodeling during labor. In sum, NF-κB plays an important role in the labor process at multiple levels by controlling inflammation and cytokines, prostaglandin synthesis, and MMP activity.

## AP-1

**Fig. 4:**
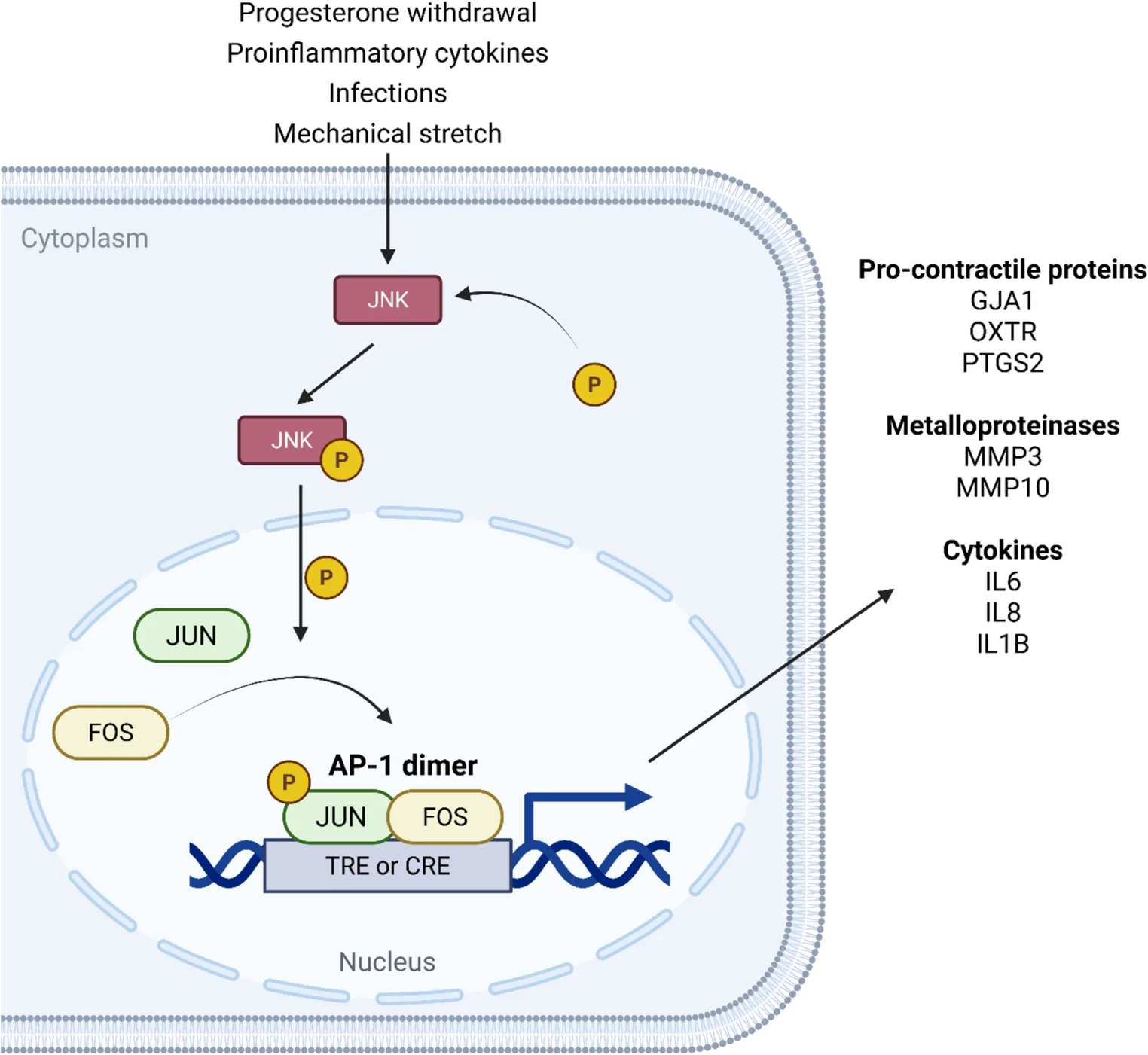
**AP-1 signaling pathway in myometrial activation. **Progesterone withdrawal, proinflammatory cytokines, infections, and mechanical stretch activate JNK, leading to phosphorylation of JUN and formation of the AP-1 JUN/FOS dimer. AP-1 binds to TRE/CRE elements in target genes to induce expression of pro-contractile proteins (GJA1, OXTR, PTGS2), metalloproteinases (MMP3, MMP10), and cytokines (IL6, IL8, IL1B), promoting myometrial activation during labor. Created with BioRender.com

The activator protein 1, AP-1 (Fig. 4) transcription factor family consists of four subfamilies: JUN, FOS, MAF and ATF [119]. These proteins share a basic leucine zipper (bZIP) domain, which mediates their dimerization, either as homo- or heterodimers, to form AP-1 complexes [120]. AP-1 transcription factors respond to a variety of stimuli, including proinflammatory cytokines, oxidative stress, and bacterial infection [120–122]. AP-1 activation involves the phosphorylation of JUN via the JUN N-terminal kinase (JNK) pathway [123]. Once activated, AP-1 dimers recognize 12-O-tetradecanoylphorbol 13-acetate response elements (TRE) as well as cAMP response elements (CRE) in target genes [124]. TRE and CRE recognition sites are present in the regulatory regions of target genes encoding CAPs in the DNA, including GJA1 and OXTR [125, 126].

Several studies investigated the role of AP-1 in preterm labor using the uterine smooth muscle, the myometrium, as a model. Throughout most of pregnancy, the myometrium is held in a quiescent state via the suppression of CAPs. It has been shown that progesterone plays a role in suppressing CAPs by interfering with AP-1 signaling [127]. Prior to the onset of labor, CAPs are highly expressed to transition from a quiescent to a contractile or active myometrium [52]. Similarly, a histone ChIP-seq analysis revealed the presence of AP-1 motifs in active regulatory regions, particularly within the putative enhancer regions of CAPs during labor [128]. Although overall FOS and JUN levels remain relatively stable throughout gestation, labor is associated with upregulation of specific AP-1 components - JUND, JUNB, and FRA2 in mice, and JUND, FRA2, and FOS in human myometrium [129]. The AP-1 complex made up of a FOS/JUN heterodimer is more stable than a JUN/JUN homodimer [120]. FOS/JUN heterodimers are potent activators of AP-1 sites. During labor, FOS/JUN heterodimers dominate as FOS expression is increased, while JUN/JUN homodimers prevail during early and mid-gestation when FOS protein levels are stably low [129]. The upregulation of these AP-1 proteins at the time of labor arises in response to hormonal and mechanical signals. Progesterone helps maintain myometrial quiescence during pregnancy, in part by suppressing AP-1 protein levels. Treatment of pregnant rats with progesterone results in the decreased expression of several AP-1 proteins, such as FRA2 and JUNB, preventing the onset of labor [130]. A study also treated pregnant rats with the progesterone receptor antagonist RU486, a model mimicking “sterile” preterm labor, which resulted in increased AP-1 protein levels and onset of preterm labor [129]. Moreover, mechanical stretch of the uterus by the growing fetus in rat models has also been shown to induce the expression of AP-1 proteins [126]. With progesterone withdrawal and mechanical stretch driving AP-1 expression near the end of gestation, genes involved in the onset of labor such as GJA1 and OXTR are transcribed. Thus, AP-1 potentially plays a role in driving preterm labor. AP-1 may also have a role during multifetal pregnancy-related preterm labor, since experiments in primary myometrial cells have shown that, upon excessive stretching, PTGS2 activity is increased via AP-1 activation, leading to an increase in prostaglandin levels [131].

In addition to pro-contractile proteins, myometrial AP-1 also controls the production of cytokines (IL6, IL8, and IL1B), matrix metalloproteinases (MMP3 and MMP10) and PTGS2 [122]. This suggests that AP-1 activation is important in infection/inflammation-induced preterm labor. In a preterm labor murine model, intrauterine injection of LPS, which mimics infection, resulted in an increase in FOS and JUN protein expression in mouse myometrium [122]. Thus, AP-1 activation occurs in association with the onset of labor in infection/inflammation-induced preterm labor mouse models.

## NFE2L2

**Fig. 5:**
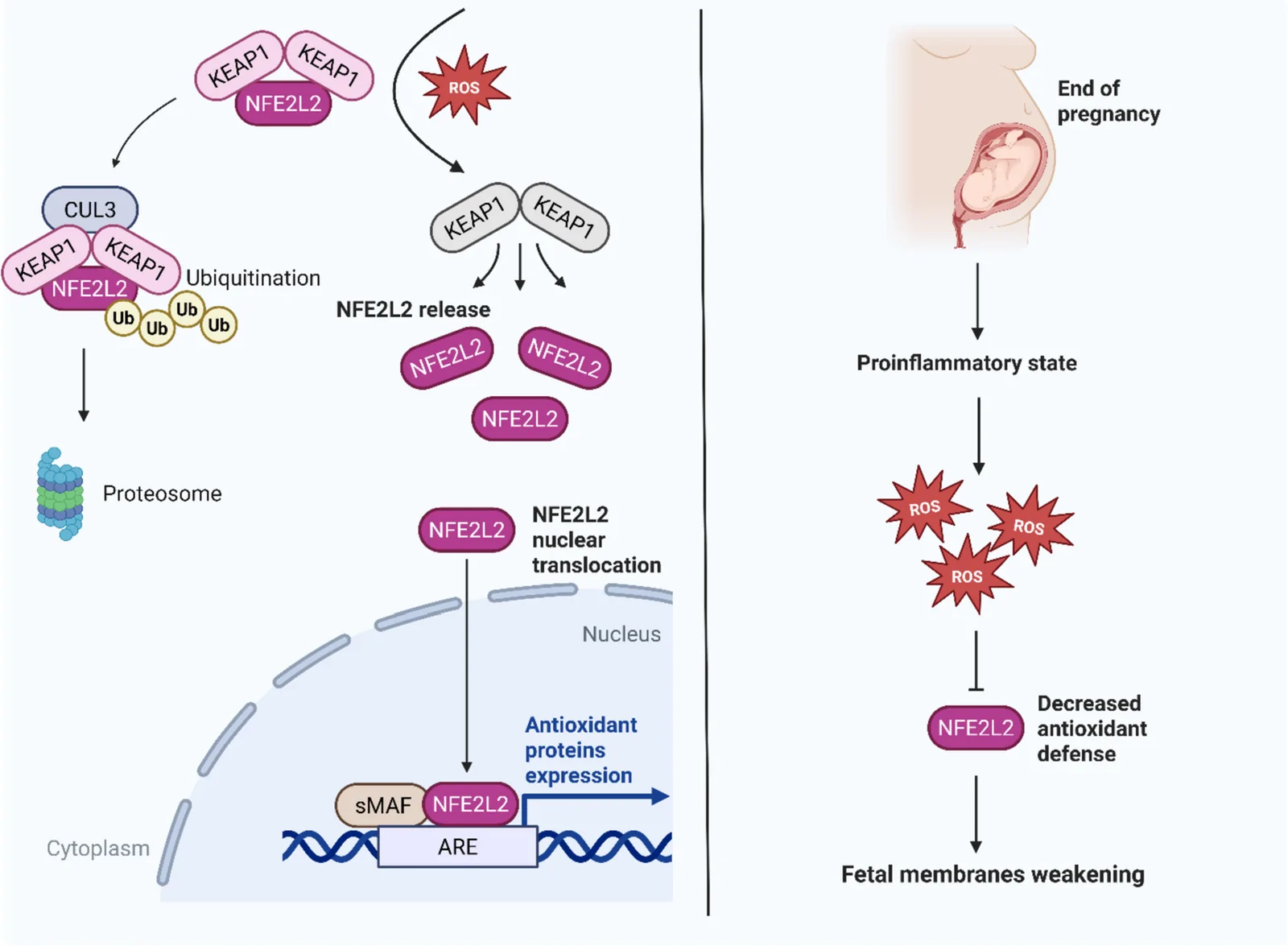
**NFE2L2 signaling pathway in oxidative stress responses during labor. **Left: Oxidative stress disrupts KEAP1‑mediated NFE2L2 degradation, allowing NFE2L2 to accumulate, enter the nucleus, dimerize with small MAF proteins, and activate antioxidant genes. Right: Near term, increased inflammation and ROS reduce NFE2L2 activity and antioxidant defenses, contributing to fetal membrane weakening. Created with BioRender.com

Nuclear erythroid 2-related factor 2, NFE2L2 (Fig. 5), also called NRF2, belongs to the cap ‘n’ collar (CNC) subfamily of basic leucine zipper (bZIP) transcription factors. NFE2L2 is primarily known for being involved in cellular defense mechanisms against oxidative stress as well as detoxification [132]. In contrast, upon constitutive activation, the transcription factor can contribute to cancer progression and resistance [133]. Under non-challenging conditions, NFE2L2 is bound and sequestered by two Kelch-like ECH-associated protein 1 (KEAP1) in the cytoplasm. KEAP1 represses NFE2L2 activity. It does so by acting as an adaptor protein for Cullin-3 (CUL3) which is responsible for the continuous ubiquitination and degradation of NFE2L2. In response to oxidative stress, KEAP1 undergoes conformational changes and releases NFE2L2 [133, 134]. Unbound NFE2L2 translocates to the nucleus where it heterodimerizes with small MAF proteins (MAFF, MAFG, MAFK). These heterodimers bind Maf recognition element (MARE), nuclear factor erythroid 2 (NFE2), antioxidant response element/electrophile response element (ARE/EpRE), and cap ‘n’ collar–small Maf binding element (CsMBE) recognition sites in target genes, with a wide variety of functions including detoxification, inhibition of inflammation, DNA damage recognition, and proteasome function [133–137].

Despite the recognized importance of immune and oxidative homeostasis in pregnancy, the precise mechanisms that prime the myometrium for labor remain incompletely understood [138, 139]. Oxidative stress and inflammation constitute the key pathways governing labor and delivery [33], but further studies are needed to identify the molecular mechanisms linking these processes. Due to its association with inflammation control and the oxidative stress response, several studies on the role of NFE2L2 in myometrial cells and the labor process have been performed. *Nfe2l2*^−/−^ mice have increased susceptibility to intrauterine inflammation, with shortened time until delivery and lower birthweight. Also, the placentas of *Nfe2l2*^−/−^ mice were shown to have increased oxidative stress and inflammatory marker levels as well as increased cell death [140]. These experiments indicate that NFE2L2 improves birth outcomes by counteracting oxidative stress and inflammation. Another study found that NFE2L2 mRNA and protein levels are lower in human fetal membranes after spontaneous term labor and delivery when compared to non-laboring fetal membranes [141]. NFE2L2 gene expression is also significantly decreased in fetal membranes obtained from women at preterm with histologic chorioamnionitis compared to those without. Transfection of primary amnion cells with NFE2L2 siRNA elicited an increase in IL6 and IL8 transcripts and release, induced by either IL1B, TNF, the TLR5 ligand flagellin or the TLR3 ligand poly(I: C) [141]. Thus, NFE2L2 exhibits anti-inflammatory properties in human fetal membranes. Stretch forces induced by the growing fetus and oxidative stress seen near the end of gestation are thought to promote a proinflammatory state which plays a role in weakening the fetal membranes. A recent study has shown that mechanical stretch in human amnion epithelial cells (hAECs) from the fetal membranes results in a significant reduction of NFE2L2 expression. This was accompanied by a proinflammatory response marked by an increase in the activation of NF-κB, ROS levels, secretion of the danger-associated molecular protein HMGB1 (high mobility group box 1) as well as LDH (lactate dehydrogenase) activity, a non-specific marker of cell stress [142]. These results support the idea that stretch can activate inflammation by inducing NF-κB and, downregulate antioxidant defense pathways by inhibiting NFE2L2, at the end of pregnancy and labor onset. Clinical studies showed that placental tissues from patients with premature and full-term ruptured membranes display increased ROS and decreased antioxidant enzyme levels [143]. NFE2L2 also plays a crucial role in maintaining mitochondria homeostasis, the main source of ROS. The levels of NFE2L2 in preterm ruptured membranes are significantly decreased, and mitochondria exhibit increased mitochondrial damage. Knockdown of NFE2L2 in hAECs isolated from the membranes results in a significant increase in ROS production and further aggravates mitochondrial damage [144]. This suggests a protective effect of NFE2L2 against ROS-induced premature rupture of membranes.

Another recent study investigated the role of NFE2L2 in regulating the inflammatory and oxidative stress responses in PHM1–31 myometrial cells in the absence and presence of the proinflammatory cytokine IL1B [145]. Treatment of PHM1-31 cells led to higher ROS levels and increased NFE2L2, and knockdown of NFE2L2 resulted in a significant increase in ROS. Downregulation of NFE2L2 led to a decrease in heme oxygenase-1 (HMOX1) and aldo-keto reductase family 1 member B (AKR1B) at the transcript and protein levels under both basal and IL1B treated conditions. NFE2L2 knockdown also resulted in reduced ferritin heavy chain 1 (FTH1) mRNA expression, but only upon IL1B exposure, while FTH1 protein is downregulated both under basal and IL1B treatment conditions. As confirmed by chromatin immunoprecipitation (ChIP) studies, NFE2L2 directly binds to the regulatory regions of these targets. As previous reports have linked HMOX1 and FTH1 to the oxidative stress response, and AKR1B1 to prostaglandin synthesis, these data demonstrate that NFE2L2 functions as a regulator of inflammatory and oxidative stress signaling as well as prostaglandin metabolism in myometrial cells [145]. A more recent study in pregnant sows identified a novel NFE2L2/MAFF signaling pathway involved in the regulation of myometrial contractility [146]. In this study, melatonin increased NFE2L2 and MAFF expression and promoted the expression of CAPs, such as OXTR and PTGS2. Functional experiments demonstrated enhanced myometrial contraction following melatonin treatment, whereas knockdown of NFE2L2 or MAFF attenuated these effects. These findings suggest that, in addition to its established antioxidant and anti-inflammatory functions, NFE2L2 may also contribute to pro-contractile transitional programs in the myometrium dependent on the cellular and physiology contact. Thus, NFE2L2 links inflammation, oxidative stress responses, and myometrial activation pathways involved in parturition, highlighting its potential as a therapeutic target for treating infection-induced preterm labor.

### MAFF

**Fig. 6:**
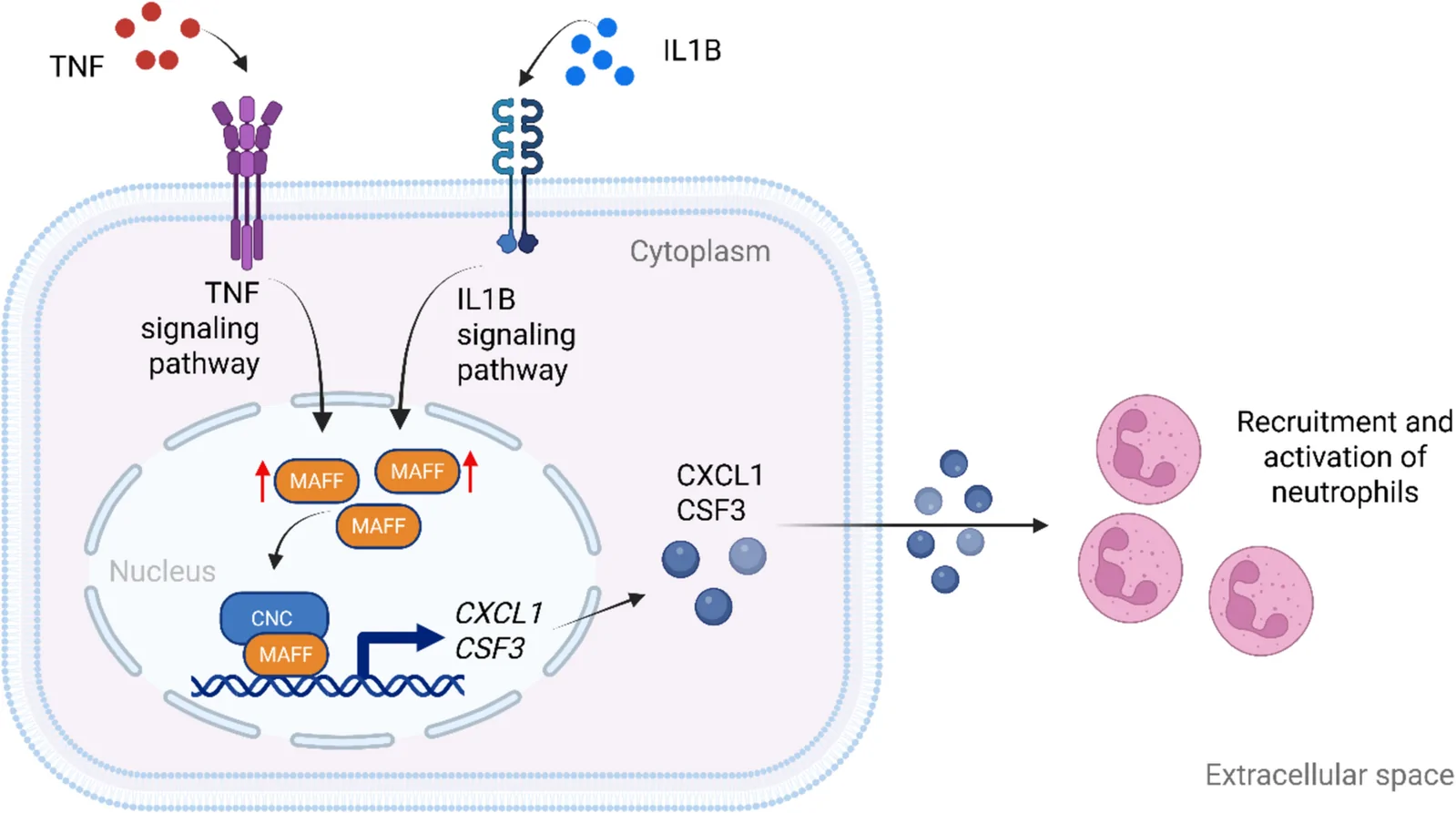
**MAFF signaling pathway in labor-associated inflammatory responses. **TNF and IL1B signaling increases MAFF expression, enabling MAFF to heterodimerize with CNC transcription factors in the nucleus and activate target genes such as CXCL1 and CSF3. The resulting chemokine secretion promotes recruitment and activation of neutrophils, contributing to the pro‑inflammatory environment associated with labor. Created with BioRender.com

The MAFF transcription factor (Fig. 6) is a member of the small MAF (avian musculoaponeurotic fibrosarcoma) basic leucine zipper (bZIP) family [147–150]. The MAFs can be categorized into two subgroups: large and small MAFs. Large MAFs contain an activation domain at the N‑terminus. They regulate gene expression in diverse cell types and tissues such as hematopoietic cells, bone, kidney, and photoreceptor cells. They are also implicated in the process of oncogenesis. The small MAFs include MAFF, MAFK and MAFG which lack a transactivation domain [147–149]. Yet, they play key roles in transcriptional regulation. Small MAF transcription factors can homodimerize as well as heterodimerize with other bZIP family transcription factors through their leucine zipper domain [148]. Small MAF homodimers act as transcriptional repressors [151]. Small MAFS heterodimerize with other bZIP transcription factors, including the cap ‘n’ collar (CNC) family members NFE2, NFE2L1, NFE2L2, NFE2L3, as well as BACH1 and BACH2. The resulting heterodimers can act either as transcriptional activators or repressors depending on their dimerization partners [148]. The small MAF homo- or heterodimer binds MARE, NFE2, ARE/EpRE and CsMBE type recognition sites on the DNA [133, 147, 150]. The small MAFs play crucial roles in controlling mammalian gene expression as they have been associated with many different pathways and/or pathologies such as cellular stress response, detoxification, diabetes, neuronal disease, and cancer.

The link between MAFF expression and myometrial cell function was established by a study reporting that MAFF is highly expressed in human myometrium at term but is not present in early gestation and in nonpregnant myometrium [152]. In later studies, it was shown that MAFF transcript and protein levels are induced by the proinflammatory cytokines IL1B and TNF in human PHM1-31 myometrial cells [153]. However, expression of the other small MAFs, MAFG and MAFK, which are highly homologous to MAFF, is not modulated by proinflammatory cytokines [154]. As proinflammatory cytokines play a major role in the onset of labor, induction of MAFF by IL1B and TNF in myometrial cells suggests a potential role for MAFF in promoting preterm labor. Knockdown of MAFF in PHM1-31 human myometrial cells resulted in a significant downregulation of CXCL1 and CSF3, both at the transcript and protein levels, suggesting that MAFF acts as a transcriptional activator controlling the expression of these genes [155]. This is of interest as CXCL1 and CSF3 have been shown to play a part in the recruitment and activation of neutrophils [156, 157], and have been reported to be significantly upregulated with spontaneous labor [158]. It was also found that MAFF knockdown in myometrial cells leads to a significant decrease in *CCL2*, *IL6* cytokine and *MMP2* and *MMP9* metalloproteinase transcripts in THP-1 monocytic cells [155], which is relevant, as CCL2 and IL6 have been implicated in labor [159, 160] and MMP2 and MMP9 contribute to cervical ripening [161].This is important as neutrophils have been found to infiltrate the uterine cervix and consequently induce labor. Moreover, the CXCL1 chemokine has been shown to induce monocyte recruitment, and the CSF3 cytokine has been shown to play a role in the maturation of both monocytes and granulocytes [162–164]. The recruitment and activation of these immune cells contribute to the proinflammatory microenvironment of the uterus at term, as well as in preterm labor. Thus, in myometrial cells, MAFF expression is controlled by proinflammatory cytokines IL1B and TNF where it acts as a positive regulator of CXCL1 and CSF3 expression. Recent evidence in pregnant sows also suggests that MAFF, together with NFE2L2, contributes to the regulation of CAP expression and myometrial contractility [146]. Given that MAFF is induced by proinflammatory cytokines that activate NF-κB and AP-1 proteins, and can cooperate with NFE2L2-dependent signaling pathways, MAFF may function as an effector that reinforces inflammatory and pro-contractile processes associated with labor.

## Transcription factors associated with myometrial quiescence during pregnancy

**Fig. 7:**
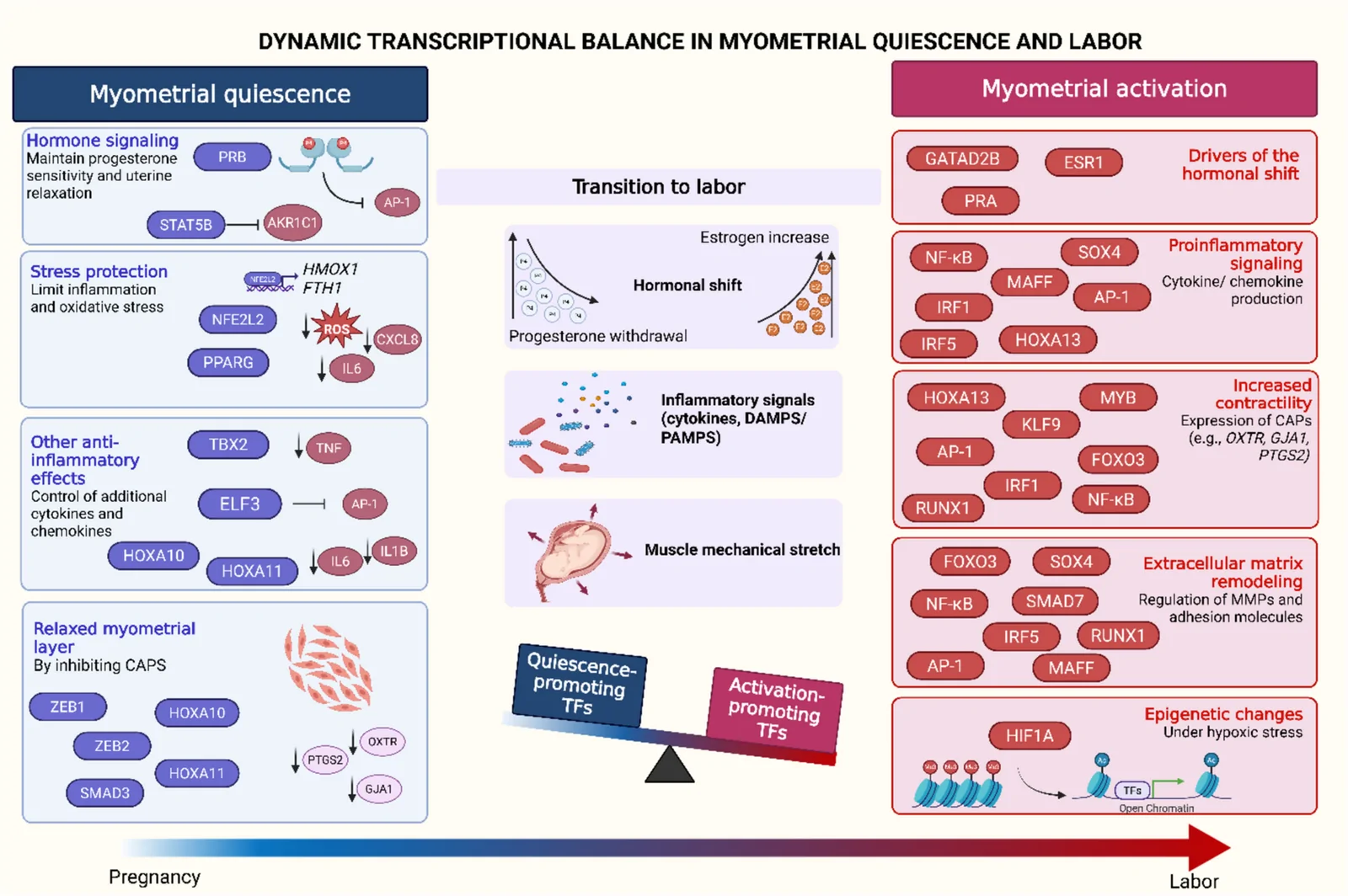
**Dynamic transcriptional networks regulating myometrial quiescence and labor. **During pregnancy, quiescence-promoting transcription factors maintain uterine relaxation by suppressing inflammatory signaling, oxidative stress, extracellular matrix remodeling, and contraction-associated proteins (CAPs). Functional progesterone withdrawal, inflammatory stimuli, and mechanical stretch promote a shift toward activation-associated transcription factors, which drive inflammatory signaling, prostaglandin synthesis, extracellular matrix remodeling, and CAP expression during labor. Together, these coordinated transcriptional programs regulate the transition from myometrial quiescence to uterine activation and parturition. Created with Biorender.com

Beyond established inflammatory and hormone-responsive transcription factors involved in labor, an increasing number of transcriptional regulators have been implicated in the control of myometrial quiescence, inflammatory activation, contractility, and tissue remodeling during pregnancy and parturition (Fig. 7). Several of these factors exhibit predominantly pro- or anti-labor functions by modulating contraction-associated proteins, prostaglandin synthesis, cytokine production, extracellular matrix remodeling, and progesterone responsiveness.

Among the transcription factors associated with uterine quiescence, the zinc finger E-box binding homeobox ZEB1 and ZEB2 act downstream of progesterone signaling in both human and mouse myometrium. Progesterone receptor activity maintains elevated ZEB1/2 expression during gestation, leading to repression of contraction-associated genes such as OXTR and GJA1 and thereby preserving a non-contractile phenotype. In parallel, ZEB1 promotes expression of the miR-199a/214 cluster, which suppresses PTGS2 expression and prostaglandin synthesis, further contributing to uterine quiescence. Near term, increased expression of the miR-200 family coordinately downregulates ZEB1/2, relieving repression of pro-contractile genes and enhancing inflammatory signaling and uterine contractility through increased PTGS2 expression [53].

Additional regulators linked to uterine quiescence include the zinc finger protein GATAD2B, which has been proposed to repress progesterone receptor expression by binding regulatory regions within the PR locus. Declining GATAD2B levels in late gestation may therefore contribute to functional progesterone withdrawal and labor initiation [165]. Similarly, overexpression of the T-box transcription factor T-box transcription factor 2 (TBX2) in hTERT-HM myometrial cells reduced TNF and interferon signaling, suggesting a potential anti-inflammatory role in the myometrium [166]. Recent evidence has also shown that the E74-like ETS transcription factor 3 (ELF3) is upregulated at term labor in human myometrial cells, functioning as a transcriptional repressor to attenuate promoter activity and counteract AP-1–mediated gene activation [167, 168]. Finally, the signal transducer and activator of transcription 5B (STAT5B) transcriptionally represses AKR1C1, an enzyme that is responsible for metabolizing progesterone [169]. AKR1C1 is elevated in the myometrium during labor [56], thus STAT5B acts as an anti-labor transcription factor.

## Transcription factors linked to myometrial cell activation during labor

Several additional transcription factors have also been linked to inflammatory amplification and tissue remodeling during labor. Increased nuclear levels of KLF5 are associated with term labor, and KLF5 silencing reduces cytokine and chemokine production, prostaglandin release, and NF-κB transcriptional activity in myometrial cells stimulated with IL1B, bacterial flagellin, or poly(I: C) [136]. Knockout of KLF9 in mice resulted in delayed parturition accompanied by altered PRA expression, NF-κB binding activity, and reduced *Oxtr* and *Crebbp* transcript levels, supporting a role for KLF family members in the regulation of the timing of labor [170]. Similarly, IRF1 and IRF5 expression increases in fetal membranes and myometrium following term labor in humans, while their knockdown in primary human myometrial cells reduces cytokines and chemokines, such as IL6, CXCL8, CCL2, IL1B, and TNF, and PTGS-driven prostaglandin production [171, 172]. Previous large-scale screening studies had identified runt-related transcription factor 1 (RUNX1) as being upregulated in laboring human myometrium [158, 173, 174]; it promotes proinflammatory cytokines, chemokines, adhesion molecules, contraction-associated mediators including OXTR and PTGFR, and the matrix-remodeling enzyme MMP9 [175]. In parallel, transcriptomic analyses identified stabilization of HIF1A in late gestation mouse placenta, associated with mitochondrial dysfunction and cellular senescence, while similar regulatory patterns were also observed in human specimens [176]. Consistently, HIF1A has been proposed as a mediator of H3K27ac histone modifications under hypoxic stress, associating it as a contractility enhancer in human myometrial cells [177].

MYB has been identified recently as an additional transcriptional regulator implicated in myometrial activation at labor. It is upregulated at term labor in human myometrial cells, as well as in a mouse model of non-infectious preterm labor, supporting its association with the transition to a contractile phenotype [167, 168]. Functionally, MYB acts as a transcriptional activator, promoting the expression of labor-associated genes such as *Gja1* and *Fos*, in part through cooperation with AP-1 transcription factors.

Recent work has also identified SOX4 as a potential regulator of labor-associated transcriptional programs in the myometrium. SOX4 expression increases during term labor and in mouse models of preterm labor, suggesting a role in myometrial activation. Mechanistically, SOX4 exerts context-dependent effects on labor-associated gene promoters through cooperation with AP-1 factors, enhancing expression of genes such as *Fos* and *Mmp11* while repressing *Ptgs2* promoter activity under specific AP-1 configurations [178].

Members of the FOXO family have also emerged as potential regulators of labor-associated inflammatory pathways in the human myometrium. FOXO1 and FOXO3 expression is increased in laboring human myometrium and is rapidly induced by IL1B stimulation in primary human myometrial cells. Functional studies demonstrated that FOXO1/3 promotes the expression of multiple pro-labor mediators, including IL6, IL8, PTGS2-derived prostaglandins, and MMP9 [179, 180]. Interestingly, FOXO3-mediated effects appeared to occur independently of NF-κB transcriptional activation, suggesting that FOXO3 may regulate labor-associated pathways through alternative transcriptional mechanisms or cofactor interactions [179].

### Transcription factor families with dual actions

While many transcription factors can have pro- or anti-labor effects, certain transcription factor families display temporally and context-dependent dual activities that integrate hormonal, inflammatory, and mechanical signals throughout pregnancy and labor. Among these, the SMAD and HOXA families represent examples of the complexity and plasticity of transcriptional regulation in the myometrium.

The SMAD (Sma and Mad-related) proteins are the principal intracellular effectors of the transforming growth factor beta (TGF-β) signaling pathway, transducing signals from ligands of the TGF-β superfamily into transcriptional responses that regulate cell differentiation, inflammation, extracellular matrix remodeling, and tissue homeostasis. SMAD proteins are divided into receptor-regulated SMADs (SMAD1/2/3/5/8), the common mediator SMAD4, and inhibitory SMADs (SMAD6/7). Through dynamic and context-dependent transcriptional regulation, SMAD signaling integrates hormonal and inflammatory cues that are central to reproductive physiology [181].

The SMAD2/3/4 proteins play a broad role in pregnancy initiation through regulating implantation, decidualization, and placental development [182, 183]. Within this framework, SMAD3 has been specifically identified as a quiescence-promoting, anti-labor regulator in the human myometrium [184]. In contrast, a shift toward inhibitory SMADs, particularly SMAD7, is associated with the transition to labor and increased inflammatory signaling [185].

In human myometrium, SMAD3 activity is reduced during labor, and its suppression enhances the expression of pro-inflammatory cytokines, chemokines, prostaglandin-synthesizing enzymes such as PTGS2, and contractility-associated mediators including PGF₂α. These findings support a model in which SMAD3 functions as a transcriptional brake limiting inflammatory- and prostaglandin-driven pathways required for myometrial activation [184].

Conversely, SMAD7 acts as a pro-labor, pro-inflammatory mediator. Its expression is increased in laboring myometrium and fetal membranes, as well as in inflammatory conditions such as chorioamnionitis. Functional studies show that SMAD7 promotes the expression of cytokines, adhesion molecules, and matrix-remodeling enzymes (e.g., MMP9), thereby facilitating membrane activation, leukocyte recruitment, and extracellular matrix remodeling. Mechanistically, these effects are linked to inhibition of SMAD2/3 signaling in primary human myometrial cells [184, 185].

HOXA transcription factors belong to the highly conserved HOX homeobox family, which regulates tissue patterning and cellular differentiation during development and adult tissue homeostasis [186]. Within the female reproductive tract, HOXA genes contribute to uterine identity and function, with persistent expression maintained in adult reproductive tissues rather than being restricted to embryogenesis [187]. In the myometrium, the best-characterized members are HOXA10, HOXA11 and HOXA13, which appear to regulate regional specialization and the balance between uterine quiescence and activation during pregnancy and labor.

Among these factors, HOXA10 and HOXA11 are predominantly associated with pregnancy maintenance and myometrial quiescence. In human term myometrium, both genes are more highly expressed in the fundal region compared with the lower uterine segment, and their overexpression suppresses the expression of inflammatory and contraction-associated genes including IL1B, IL6, GJA1, and PTGS2 in human myometrial cells [188]. These findings suggest that HOXA10 and HOXA11 contribute to maintaining a less contractile and less inflammatory phenotype in the fundus during pregnancy. In addition, HOXA10 expression in human myometrium is hormonally regulated, as progesterone represses HOXA10 expression in primary myometrial cultures, linking this factor to progesterone-dependent uterine quiescence mechanisms [189]. Consistently, alterations in progesterone receptor signaling during labor have been associated with changes in HOXA10 expression in human myometrium [60].

In contrast, HOXA13 appears to promote a more activated and region-specific myometrial phenotype. Studies using human pregnant myometrial tissues and cultured myometrial cells demonstrated that HOXA13 is enriched in the lower uterine segment before labor and regulates genes involved in cell adhesion, prostaglandin signaling and inflammation, including prostacyclin synthase (PTGIS), and periostin (POSTN) genes [190]. Functional studies further showed that HOXA13 increases myometrial cell contractility and adhesion, while also stimulating IL1B production and downstream induction of contraction-associated proteins such as PTGS2, GJA1, and OXTR [191]. Mechanistically, IL1B signaling was identified as a key mediator of these effects, since IL1 receptor blockade attenuated HOXA13-induced inflammatory and contractile responses. HOXA13 also promoted recruitment of monocyte-like THP-1 cells [191]. Therefore, unlike HOXA10 and HOXA11, HOXA13 displays predominantly pro-inflammatory and pro-contractile functions in human myometrium.

Evidence for HOXA-mediated regulation of pregnancy and labor derives mainly from human tissues, primary human myometrial cells, and human myometrial cell lines. However, animal models also support an important role for HOXA genes in uterine biology. HOXA10- and HOXA11-deficient mice exhibit profound reproductive defects and impaired uterine differentiation, highlighting their importance for pregnancy establishment and maintenance [192–194]. In rats, altered *Hoxa10* expression has been associated with abnormal myometrial adaptation and premature activation signatures during pregnancy [195]. Collectively, these studies support the concept that HOXA transcription factors participate in the regional and functional specialization of the pregnant myometrium, with HOXA10/11 favoring quiescence and HOXA13 promoting inflammatory and contractile pathways associated with labor onset.

### Conclusions and outlook

There is still much to learn about the mechanism contributing to preterm and term labor in humans. Some of the described studies on the role of transcription factors in the myometrium and uterine contractions are correlative, while others provide mechanistic insights. Together, these studies reveal a complex regulatory network by a multitude of transcription factors governing pregnancy, myometrial cell function, labor, and parturition. This research provides compelling evidence that transcription factors PR/ER, NF-κB, AP-1, PPAR, MAFF, and NFE2L2 play critical roles in various facets of myometrial cell biology and in the onset of labor. In addition, a growing number of emerging regulators, including ZEB1/2, GATAD2B, TBX2, ELF3, STAT5B, KLF5/9, IRF1/5, RUNX1, HIF1A, MYB, SOX4, and FOXO1/3, further highlight the complexity of transcriptional control in the pregnant uterus. Moreover, transcription factor families such as SMAD and HOXA illustrate how transcriptional regulation during pregnancy and parturition is highly dynamic and context-dependent, with individual family members exerting distinct or even opposing functions depending on hormonal, inflammatory, spatial, and temporal cues. Clearly, further investigation to unravel the respective roles of these transcription factors in the labor process is warranted.

Deepening our understanding of the molecular mechanisms in which these transcription factors act in the context of preterm and term labor will help to potentially improve birth outcomes. This knowledge could be greatly beneficial in developing novel therapeutic strategies to prevent and overcome the risks and complications associated with preterm labor. One promising option involves cell-penetrating peptides (CPPs), which can function as vectors capable of crossing cell membranes. For example, CCP conjugated to an IKK inhibitory peptide, called Pen-NBD, blocked PTGS2 induction by IL1B in primary myometrial cells. In the same study, a small molecule inhibitor, Sc514, targeting the IKK complex in the NF-kB pathway, was also able to diminish uterine inflammatory gene expression [196]. Similarly, dietary flavonoids, luteolin and kaempferol, inhibited IL1B-induced RELA DNA binding in human myometrial cells and have been proposed as a therapeutic approach to prevent preterm labor [197]. With respect to AP-1 proteins, high levels of JUN, FOS, and FOSL2 have been observed in women undergoing caesarean section with uterine contractions. These AP-1 proteins have been proposed as possible biomarkers for labor timing [198]. Moreover, the progesterone receptor mediates the effects of progesterone, maintaining pregnancy by suppressing uterine contractility. In most mammalian species, progesterone levels are decreased at the onset of contractions, however in humans, labor is thought to be initiated by a functional progesterone withdrawal. Thus, non-metabolizable progestogens might represent a novel therapeutic tool to counter preterm birth [52, 58, 199]. In addition, the anti-inflammatory effects of PPARG make it a possible target for controlling the labor process. Concomitantly, activation by the PPARG agonist rosiglitazone inhibited inflammation and reduced preterm labor in a mouse model in association with an upregulation of NFE2L2 and HMOX1 [91]. Likewise, MAFF is a transcription factor that regulates the expression of genes involved in the inflammatory response, by controlling the expression of cytokines and chemokines in myometrial cells. Thus targeting MAFF is a viable option for controlling the onset of contractions [155]. As for NFE2L2, which controls both the oxidative stress response and AKR1B1-mediated prostaglandin metabolism, targeting this pathway may offer a dual mechanism for reducing labor onset. In support of this, pharmacological activators of NFE2L2, such as sulforaphane and bardoxolone methyl, have demonstrated efficacy in enhancing NFE2L2 activity and reducing ROS levels in preclinical models [200]. Finally, the IRF1 transcription factor has been implicated in promoting preterm labor and IRF1 inhibitors could potentially act as therapeutics for managing preterm labor [171].

The control of the labor process is complex as the same transcription factor may have opposing functions depending on the context. It is important to carry out highly controlled experimental and preclinical studies. The data presented here showcases that maintenance of pregnancy and the initiation of labor are mediated by many interconnecting transcription factor pathways. Further research must be conducted to comprehend how these are specifically involved in preterm labor and to develop novel therapeutic strategies to diagnose, prevent, or delay preterm labor.

## Data Availability

Not applicable.
